# Reply to Pennington, T.E.; Thompson, J.F. Sentinel Node Biopsy in Melanoma Remains a Valuable Clinical Tool. Comment on “Dixon et al. Primary Cutaneous Melanoma—Management in 2024. *J. Clin. Med.* 2024, *13*, 1607”

**DOI:** 10.3390/jcm14010216

**Published:** 2025-01-02

**Authors:** Anthony Joseph Dixon, Michael Sladden, Christos C. Zouboulis, Catalin M. Popescu, Alexander Nirenberg, Howard K. Steinman, Caterina Longo, Zoe Lee Dixon, Joseph Meirion Thomas

**Affiliations:** 1 Department of Research, Australasian College of Cutaneous Oncology, Docklands, VIC 3008, Australia; alexander.nirenberg@gmail.com (A.N.); zoedixon96@gmail.com (Z.L.D.); 2 Research, American Osteopathic College of Dermatology, Kirksville, MO 63501, USA; 3 Department of Dermatology, University of Tasmania, Launceston, TAS 7005, Australia; m.sladden@doctors.org.au; 4 Departments of Dermatology, Venereology, Allergology and Immunology, Staedtisches Klinikum Dessau, Brandenburg Medical School Theodor Fontane and Faculty of Health Sciences Brandenburg, 06847 Dessau, Germany; christos.zouboulis@mhb-fontane.de; 5 Department of Dermatology, Carol Davila University of Medicine and Pharmacy, 020021 Bucharest, Romania; catalin.m.popescu@gmail.com; 6 Department of Surgery, Campbell University, Buies Creek, NC 27506, USA; hksteinman@gmail.com; 7 Department of Dermatology, University of Modena and Reggio Emilia, 41121 Modena, Italy; caterina.longo@gmail.com; 8 Department of Dermatology, Centro Oncologico ad Alta Tecnologia Diagnostica, Azienda Unità Sanitaria Locale—IRCCS di Reggio Emilia, 41121 Reggio Emilia, Italy; 9 Better Rehab, Occupational Therapy, Surrey Hills, Melbourne, VIC 3127, Australia; 10 Department of Surgery, Formerly of Royal Marsden Hospital, London SW3 6JJ, UK; meirion.thomas@outlook.com

1. We note with interest the commentary by Pennington and Thompson (P&T) regarding our detailed update on the management of primary cutaneous melanoma in 2024 [1].
2. In many ways their commentary reminds us of the writings of Eaglstein. His practice changing research on the benefits of occlusive dressings was first published in 1984 [2]. In 1993, 2001, and again in 2010, he published concerns about the time lags between the reporting of his evidence-based findings and their general adoption by physicians [3,4,5].
3. In a similar fashion, P&T are correct in stating that some of our management plans do not align with “current practices worldwide”. However, current practices and guidelines can often be out of date and are sometimes based on limited data available at the time. Hopefully our research will result in guidelines being modified.
4. P&T advocate usage of the Melanoma Institute Australia’s Sentinel Node Metastasis Risk Calculator, created by Thompson and colleagues (MIA tool) and available at https://www.melanomarisk.org.au/SNLLand (accessed on 24 December 2024). The MIA tool is not and should not be included in any melanoma guidelines, as it results in excessive treatment of young melanoma patients and leaves high-risk patients without access to beneficial treatments.
5. Melanoma surgeons may struggle to accept that science has moved on from the outdated SLNB assertions, many of which are perpetuated in the P&T commentary. It is understandable that some clinicians would find it difficult to accept that some of their past management interventions are now known to be suboptimal. As science moves on, so must clinical practice.
6. Perhaps most surprising is that several of the disproven assertions made by P&T were studied and found false by research in which Thompson was a co-author [6,7]. Any suggestion in 2025 that there is some therapeutic gain from SLNB is a clinging to misconceptions and denying current evidence.
7. This is evident in P&Ts usage of the inaccurate and misleading term “micrometastases”. Removing these so-called micrometastases does not affect survival. Removing all adjacent nodes also does not improve survival [6,7]. Further, there is increasing evidence that positive nodal involvement in younger patients is indeed evidence of the vital physiological role the nodes are playing in disease management [8]. All SLNB can do is provide prognostic information. It is a test and not a therapy. As such, we use the appropriate terms positive or negative in describing SLNB results.
8. The recent research identifying the substantial role of patient age in melanoma management is also apparently poorly appreciated by P&T. As we have identified, mortality substantially increases with advancing age, whilst SLNB positivity rates are dramatically lower in older patients from immunosenescence [8].
9. P&T question our methodology and suggest we identified only two theoretical examples. This is not the case. We analysed data from El Sharouni [9] and Lo [10], both large studies endorsed by P&T. We evaluated the implications of SLNB positivity on mortality for all age groups, Breslow thicknesses, melanoma subtypes, body sites both sexes, with or without ulceration. Indeed, we analysed thousands of scenarios in the analysis that lead to our study on age and melanoma [8].
10. The discussion by P&T of two scenarios misrepresent our findings and ignore or distort the current knowledge of SLNB in managing melanoma.
11. Their first example is a 20-year-old female with a 0.4 mm ulcerated melanoma. The long-term melanoma-specific mortality risk (MSMR) is between 0.6% and 1.3%, depending on the subtype of melanoma and body location [11]. Yet their MIA tool assesses this patient as having a 12% risk of SLNB positivity [12].
12. P&T then indicate that should this 20-year-old have a positive SLNB, adjuvant drug therapy (ADT) or participation in a clinical trial might be advisable. The specificity of SLNB in predicting mortality in this circumstance is under 10%. P&T are hence advocating SLNB for 100 patients to potentially offer 12 patients who are SLNB-positive ADT or participation in a drug trial. Yet only 1 of these 100 patients could stand to benefit, given their approximately 1% mortality risk. Our view is that such practice is excessive and unwarranted based on 2025 scientific knowledge. The net potential harm, including from the adverse events of SLNB and ADT, substantially outweighs any potential overall benefit. P&T claim there are multidisciplinary melanoma units that would engage such a clinical practice. We would hope such is no longer the case in 2025.
13. The other scenario P&T focus on is an 80-year-old male with an intermediate thickness melanoma. P&T argue that it is not appropriate to offer all such patients ADT when 33% would be expected to die of their melanoma. We have identified that most deaths from melanoma in this age group are associated with negative SLNB tests [8].
14. P&T hence advocate that interventions including SLNB and ADT are justified when 1% of 20-year-olds might benefit but are concerned that only 37% of 80-year-old patients might need these interventions. This demonstrates a denial of the science for P&T to conclude that needlessly subjecting 99% of patients to unnecessary interventions is perfectly appropriate but needlessly subjecting 63% is unacceptable. Perhaps more than any other aspect of their commentary, this strongly suggests that P&T are failing to accept or are ignoring the science of melanoma management in 2025. We invite P&T to reconsider.
15. P&T misquote us in many sections. We do not advocate Berlin Ultrasound for melanoma patients. We point out that at the very best, it can be just as accurate and, hence, just as ***inaccurate*** as SLNB. Neither test is justified for most melanoma patients.
16. The BAUSSS biomarker is the single most accurate tool in predicting melanoma survival at present in clinical practice [13]. It is especially important and useful because it comes at no added cost, hospitalization, morbidity, anaesthesia, or surgery to the patient or the health system. All the information is readily available from the patient’s medical record and the pathology report of the primary melanoma. This information, combined in a multivariate approach, provides a “C” score of over 0.73.
17. P&T acknowledge that a “C” score of 0.7 is a “good model”. They state that a “C” score of 0.8 is excellent. There is no current tool, operation, or blood test pertaining to melanoma, alone or in combination, that provides a “C” score of 0.8.
18. In contrast, SLNB has a “C” score of 0.6. This is of value but is not competitive with BAUSSS. Indeed, when SLNB is combined with BAUSSS, the improvement in “C” score is only in the order of 3%, with confidence limits frequently overlapping [14]. P&T state that this 3% increase is substantial. It is difficult to understand why an improvement in a “C” score from 0.73 to 0.76 could be regarded as “substantial”.
19. Furthermore, no current melanoma guidelines include protocols based on BAUSSS plus SLNB status. P&T advocate such an approach yet criticise clinicians who do not follow guidelines. We are not aware of any multidisciplinary melanoma unit that incorporates this “substantial” approach. How “substantial” can it be?
20. With such a disappointing 0.6 “C” score to predict mortality, and contrary to the suggestion of P&T, SLNB status does not suitably identify patients who may benefit most from adjuvant drug therapy (ADT).
21. P&T comment on the option of immediate wide local excision (WLE) when the clinical diagnosis of a thin melanoma is confident. This practice is commonplace in Europe. Contrary to their assertion, skilled dermoscopists are remarkably accurate at identifying primary melanoma including assessments of thin, intermediate, and thick tumours [15,16,17,18]. P&T incorrectly underplay the sophistication of current dermoscopy usage in the dermatology world today.
22. In this context, P&T repeat another dated claim that SLNB must be performed at the same time as WLE to maximise SLNB accuracy. They argue that, amongst other concerns, a delayed SLNB undertaken after WLE is compromised. The evidence suggests otherwise [19,20]. Regardless, this is a moot point, given that SLNB would now be uncommonly considered.
23. Melanoma science and management has moved on from the unfulfilled hopes for SLNB of the 1990s. We have known since 2014 that SLNB does not improve melanoma-specific survival (MSS) [7]. We have known since 2017 that removing nodes adjacent to a positive sentinel node does not improve MSS. We now know how substantial patient age is at altering melanoma outcomes^8^ and how SLNB trends with age are the reverse of survival trends [8].
24. We do not yet know if SLNB compromises overall survival through such risks as thromboembolic disease, because the investigators of the key trials have still not published such data. This is so despite overall survival being the primary ethics approved endpoint of the MSLT1 trial [7,21].
25. P&T claim that SLNB “is widely accepted as safe, effective and valuable tool”. We know of no achievement of the SLNB tool other than a narrow refining of mortality prediction that may be of limited assistance to primary cutaneous melanoma patients between 40 and 60 years of age. Yet the adverse events associated with SLNB and the erroneous and deleterious implications of linking SLNB positivity to access for ADT are far from safe. This is a further example of the term “safe and effective” being misleadingly claimed and used regarding health interventions [22,23].
26. We know that the traditional TMN staging system is now substantially limited compared to BAUSSS. TMN only categorizes Breslow thicknesses into four broad levels: under 1 mm thick, 1 mm to 2 mm thick, 2 mm to 4 mm thick, and over 4 mm thick. There is a further classification of Breslow being under 0.8 mm when a tumour is not ulcerated. Site, subtype, and sex are not factored. But most importantly, age, the second most important risk factor in assessing survival determination, is not even a component of the outdated and often unhelpful TMN staging tool [14].
27. For example, a 20-year-old female patient with a non-ulcerated 2.1 mm Breslow thickness superficial spreading melanoma on her arm has a long-term MSMR of around 4%. An 80-year-old male with a 4 mm desmoplastic non-ulcerated melanoma on the scalp has a MSMR of around 40%. Yet both patients are staged as Tlla (T3a) under the TNM staging tool.
28. It is time that the overly optimistic hypotheses and mythology long associated with SLNB are superseded by evidence-based medicine. Our melanoma patients deserve up to date, optimum management.
